# Evaluation of Motor Neuron Excitability by CMAP Scanning with Electric Modulated Current

**DOI:** 10.1155/2015/360648

**Published:** 2015-08-26

**Authors:** Tiago Araújo, Rui Candeias, Neuza Nunes, Hugo Gamboa

**Affiliations:** ^1^Department of Physics, Faculty of Sciences and Technology, New University of Lisbon, Lisbon, Portugal; ^2^PLUX Wireless Biosignals S.A., Lisbon, Portugal

## Abstract

*Introduction*. Compound Muscle Action Potential (CMAP) scan is a noninvasive promissory technique for neurodegenerative pathologies diagnosis. In this work new CMAP scan protocols were implemented to study the influence of electrical pulse waveform on peripheral nerve excitability. *Methods*. A total of 13 healthy subjects were tested. Stimulation was performed with an increasing intensities range from 4 to 30 mA. The procedure was repeated 4 times per subject, using a different single pulse stimulation waveform: monophasic square and triangular and quadratic and biphasic square. *Results*. Different waveforms elicit different intensity-response amplitude curves. The square pulse needs less current to generate the same response amplitude regarding the other waves and this effect is gradually decreasing for the triangular, quadratic, and biphasic pulse, respectively. *Conclusion*. The stimulation waveform has a direct influence on the stimulus-response slope and consequently on the motoneurons excitability. This can be a new prognostic parameter for neurodegenerative disorders.

## 1. Introduction

The Compound Muscle Action Potential (CMAP) scan is a noninvasive diagnosis technique for neurodegenerative pathologies, such as Amyotrophic Lateral Sclerosis (ALS). It allows a quick analysis of the muscle action potentials in response to motor nerve stimulation, by electrical stimulation applied on the surface of the motor nerve and response evaluation by surface Electromyography (sEMG) at muscle level. Each motor unit (MU) of muscles has a different stimulus intensity (SI) at which it is activated, meaning that MUs have different thresholds. Varying the intensity of the stimuli applied, gradually increasing from subthreshold to supramaximal values, will sequentially activate all MUs in the muscle. This way, it is possible to obtain a graphical representation of the evoked action potentials amplitude in the muscle versus the stimulation intensity. This record will show a sigmoid tendency which is called the CMAP scan [1–3].

Henderson et al. [4] examined the variability of the CMAP scan between healthy and ALS subjects. Evidences were found for a significant difference in relation to CMAP scan evolution, number of steps (visible jumps in CMAP amplitude within consecutive stimuli), and size, since ALS patients present more and larger steps on the stimulus-response curve than healthy controls. A healthy CMAP scan has traditionally a sigmoid tendency curve for the Motor Unit Action Potential amplitude in response to a linear increase of current intensity. This technique provides clinically relevant information about reinnervation processes, number and size of functional motor units, and neuromuscular activity/excitability. Moreover, CMAP amplitude has significant correlation with muscle strength, motor unit number estimation, and functional disability in ALS [5].

To be used as a clinical tool, stimulation parameters must be standardized and quantified in order to enable uniform collection and comparison of data. Several studies have been made recently to verify the potentiality of this technique, investigating the influence of different parameters in the quality of the CMAP scan [1]. A consensual way is based on the analysis of key points like the maximum CMAP amplitude, S5, S50, and S95 (the stimulus intensity that elicited 5%, 50%, and 95% of the maximum CMAP amplitude, resp.), SI range (the difference between S95 and S5), and step percentage [1], despite the fact that the physiological influence of different stimulation parameters remains unknown.

An untested parameter of the CMAP is the waveform of the electrical pulse used in the nerve stimulation. The waveform is a graphical representation over time of a signal, as it reflects its shape. In electrical stimulation, the waveform represents the variation over time of the applied current or voltage on the muscle or nerve. It can be monophasic or biphasic and also have different shapes like sine and square, among others.

This work aims to study the influence of different pulse modulated waveforms in peripheral nerve excitability, by CMAP scan technique, on healthy subjects for future comparison in ALS patients. To accomplish that, an electrical stimulation protocol was developed and biosignals from healthy subjects were acquired, analyzed, and processed, in order to extract features for the analysis of the different waveforms' influence in the stimulation of the peripheral nerve. The waveforms tested in the stimulation protocol were monophasic square, triangular, and quadratic waves, and also a biphasic square wave.

## 2. Methods

### 2.1. Subjects

In this study a total of 13 healthy subjects were submitted to the same test. This group was composed of 7 males and 6 females, mean age of 26.00 (3.63) years, range from 20 to 36 years, and without clinical history on neurologic disorders. All subjects with predisposition to peripheral nerve problems were excluded from the study. This study protocol was approved by the local ethics committee.

### 2.2. Instrumentation

**Figure 1:**
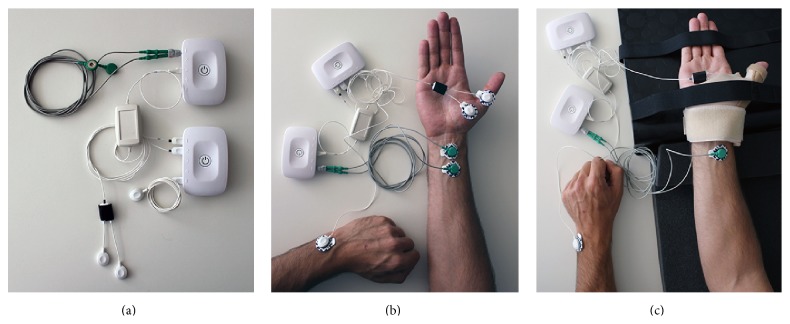
Illustration of the equipment used and electrodes placement. (a) The electrical stimulator and biosignal acquisition unit. (b) Electrodes placement: stimulation electrodes on the median nerve, sEMG electrodes on the thumb, and ground electrode on the wrist. (c) Hand fixation for the test.

The stimuli were applied in the median nerve on the right wrist and EMG signal was collected on the Abductor Pollicis Brevis muscle surface on the right thumb. Self-adhesive pregelled Ag/AgCl, 30 × 24 mm disposable electrodes were attached to the skin for acquisition and stimulation and placed according to SENIAM [6] standards. The ground electrode was placed on the left wrist ulnar styloid process. During the test, the subjects were seated, motionless and relaxed, with thumb fixation to minimize movement artifacts.  Figure 1 illustrates the equipment and electrodes placement for the protocol applied.

### 2.3. Stimulation and Acquisition

**Figure 2:**
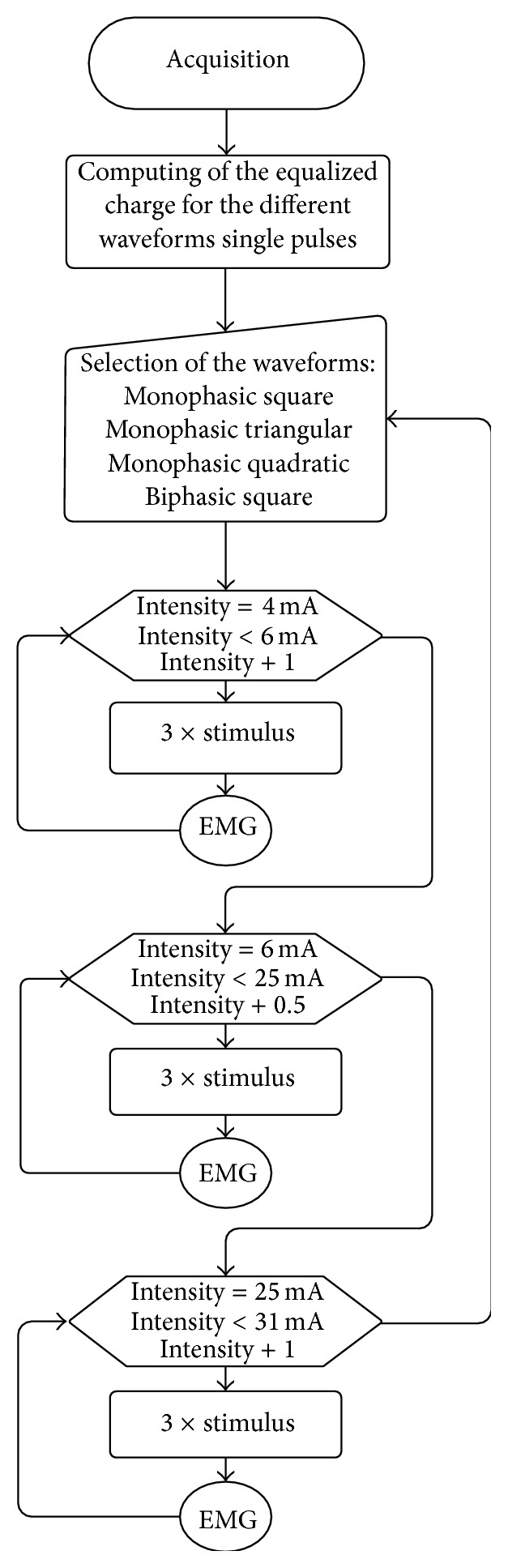
Flowchart of the stimulation protocol and biosignals acquisition steps.

Stimulation was performed with increasing intensities range from 4 to 30 mA. Different number of stimuli and current increment steps were tested in order to have a stimulation protocol that would allow obtaining a curve with enough resolution but not excessive test duration. After extensive testing, it was perceived that only 3 stimuli per step were sufficient to have high resolution in the CMAP. To optimize the relation between curve definition and test duration, the current increment steps have different values depending on the correspondent curve zone. This way, a small step increment of current intensity is defined to increase the definition of the sigmoid curve only on the transition zones. On the initial (4 to 6 mA) and final (25 to 30 mA) stages the current increment would be of 1 mA for each step. From 6 to 25 mA the increment was 0.5 mA. To properly cancel noise and mechanical artifacts, the pulses were randomly distributed in frequencies from 1.8 to 2.2 Hz. The EMG signal was acquired with a 3000 Hz sampling frequency, amplified with a gain of 201 and 12 bits of resolution. A combined wireless, miniaturized and synchronized unit was specifically developed for EMG acquisition and nerve stimulation [7, 8]. The system was previously validated in comparison with a clinical system installed in Hospital Santa Maria, Lisbon. The acquisition protocol is illustrated in the flowchart (Figure 2). The procedure was repeated 4 times per subject, each repetition using a different single pulse stimulation waveform. A standard square wave was applied in test 1, a triangular wave in test 2, and a quadratic wave in test 3. In all these 3 protocols, monophasic single pulses were used with the same intensities. A 4th protocol was tested with a biphasic single pulse square wave, with the same intensities of the other tests.

The current charge difference from each waveform was taken into account in the data analysis. The stimulation charge was computed according to the stimulation intensity and waveform, based on the following formula:

( 1 ) $$\begin{array}{c} Q=\overset{t_{2}}{\underset{t_{1}}{\int }}I\;dt, \end{array}$$

where *I* represent the current intensity and *t*_1_ to *t*_2_ is the stimulus time range. The different single pulses total charge has been equalized for each current intensity, maintaining the amplitude and varying the pulse-width time. From the theory, the total charge of the electrical impulse is the most reliable measurement of the biological reaction to the electrical stimulation [9].

### 2.4. Processing

The collected biosignals were processed and features were extracted, according to the following steps:

1. Detecting peak-to-peak amplitude of the stimulus-response M-wave.
2. CMAP scan composition via interpolation.
3. Extracting S5, S50, S95, and SI range and stimulus-response amplitude elicited by these parameters.
4. Detecting the beginning (sigmoid trigger intensity), final (sigmoid plateau first intensity), and slope of the resulting sigmoid.
5. Computing the intersubject mean and standard deviation of the computed parameters.
6. Computing the differences in the computed parameters for each waveform, when comparing with the traditional square wave.

These steps were repeated for each waveform type and all subjects. Data analysis processing scripts were implemented in Python. All data had posterior visual validation by two specialists.

## 3. Results

Each subject was analyzed regarding the maximum CMAP amplitudes, the excitability parameters (S5, S50, and S95, stimulus current intensity in mA that elicited correspondent response amplitude in mV), the sigmoid slope, and current intensity differences of the CMAP scan, between each different waveform.

**Figure 3:**
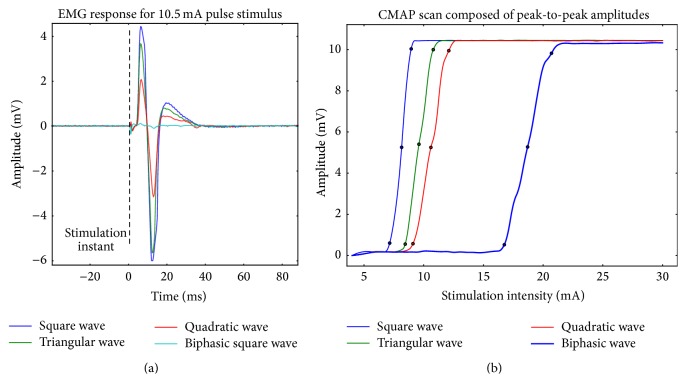
(a) CMAPs acquired in a fixed intensity step for each waveform: 10.5 mA. Differences in the waves' amplitude, with the same intensity stimulation, can be observed. (b) Four CMAP scans generated with four waveforms, for one example subject.

Figure 3(a) presents a CMAP wave elicited by the four different waveforms with the same pulse amplitude (10.5 mA). Differences in the responses' peak-to-peak amplitude can be observed, using the same current intensity and charge.  Figure 3(b) shows, for one example subject, four CMAP scans generated with four waveforms. Like in Figure 3(a), the differences between the waveforms in the stimulation intensities to generate the same response amplitude can be observed.

Table 1 presents the mean CMAP scan sigmoid slope differences between each waveform in comparison to the square wave. The value is given in percentage, assuming that the square wave's sigmoid slope is 100%.

In Table 2 the mean intensity differences regarding the stimulation parameters S5, S50, and S95 between each waveform and the square wave are presented. As the stimulation parameters increase in amplitude (from S5 to S50 and S50 to S95), the difference between each wave (from square to triangular, triangular to quadratic, and quadratic to biphasic square) also increases. This is highly correlated with the slope of each wave.

## 4. Discussion

There are several theoretical models to describe the electrical properties of the biological tissue. However, there are also several constraints which cannot be simulated, like the patient comfort to some type of stimulation, the nervous fiber fatigue associated with the habituation to the electrical pulse, or the possible changes of tissue impedance during stimulation (increase of temperature, increase of skin sudation, microirritations to the tissue after consecutive stimulus application, etc.). Some effects are also impossible to contemplate in a theoretical model, such as the study in injured patients or with an early diagnosis for the disease.

Peripheral nerve stimulation is influenced by external variables that are hard to control, like the adipose tissue layer that the electrical current has to pass, the distance between the stimulation electrodes and the nerve to be stimulated, among other body inhomogeneities. Taking this into consideration, the chosen analysis parameters were the ones that allowed a more objective assessment of the considered effects among different subjects.

The results show that the square pulse, besides needing less current intensity to generate the same response amplitude as the other waves, is also the one that presents a steeper curve slope. This means that, for the square wave, the intensities range between the beginning and final of the CMAP scan is usually shorter than for the other waves.

The quadratic wave, among the monophasic waves group, represents the stimulation pulse that needs a larger current intensity to elicit the same response amplitude in comparison with the other waves. This fact consequently translates into an inferior sigmoid slope. This happens possibly due to a nervous fiber sensibility to charge transfer rate, since in the used setup all single pulses have the total charge equalized.

Concerning the biphasic square pulse, it is possible to verify that it has a distinct behavior from the monophasic pulses, with activation intensities of the response levels S5, S50, and S95 quite superior and also a rather inferior sigmoid slope. This indicates that, possibly, only one of the flanks of the biphasic waveform is activating the nerve fibers. The monophasic waveforms have a more linear behavior, while the biphasic waveform presents a more unstable behavior with greater variations.

The analysis of the influence of the waveform on the peripheral nerve stimulation reveals new effects in the context of the nerves' excitability. The control of this parameter allows varying the stimulus-response curve slope.

Electrophysiology studies reveal significant differences between healthy subjects and neurodegenerative patients. Upon this, when a variable that is able to manipulate or change the sensibility of the CMAP stimulus-response curve is introduced, a question arises: Will this variable have the same behavior when evaluating patients? How about in subjects with a prior propensity to develop the pathology? Is it possible that subjects with pathology or propensity to develop will show differences in the sensibility to the electrical charge? These are the relevant questions that this work starts to address, studying healthy subjects.

This study is now being prepared to be implemented on ALS patients. The evaluation of new wave parameterizations in healthy subjects, in addition to being a basis for comparison for future patients evaluation, also represents a contribute for research associated with the CMAP scan methodology and a step forward on the understanding of the electrical pulse waveform influence on peripheral nerve excitability.

## Figures and Tables

**Table 1:** Mean CMAP scan slope differences in comparison to the standard square wave. The value is given in percentage, assuming that the square wave's sigmoid slope is 100%.

Slope differences	Mean	SD
Square-triangular	81%	11%
Square-quadratic	67%	11%
Square-biphasic	44%	19%

**Table 2:** Waveforms current intensity differences.

Intensity differences (mA)	S5 Mean (SD)	S50 Mean (SD)	S95 Mean (SD)
Triangular-square	1.22 (0.57)	1.60 (0.63)	1.92 (0.89)
Quadratic-square	1.82 (0.70)	2.44 (0.50)	3.01 (0.81)
Biphasic-square	5.39 (2.41)	7.48 (2.27)	8.62 (2.79)
